# Endodontic Management of a Severely Dilacerated Mandibular Third Molar: Case Report and Clinical Considerations

**DOI:** 10.1155/2018/7594147

**Published:** 2018-10-08

**Authors:** Suraj Arora, Gurdeep Singh Gill, Priyanka Setia, Anshad Mohamed Abdulla, Ganapathy Sivadas, Vaishnavi Vedam

**Affiliations:** ^1^Department of Restorative Dentistry, College of Dentistry, King Khalid University, Abha, Asir Province, Saudi Arabia; ^2^Department of Conservative Dentistry & Endodontics, JCD Dental College, Sirsa, Haryana, India; ^3^Department of Pediatric Dentistry and Orthodontic Sciences, College of Dentistry, King Khalid University, Abha, Asir Province, Saudi Arabia; ^4^Department of Pedodontics and Preventive Dentistry, Faculty of Dentistry, Asian Institute of Medicine, Science and Technology (AIMST) University, Kedah, Malaysia; ^5^Department of Oral Pathology, Faculty of Dentistry, Asian Institute of Medicine, Science and Technology (AIMST) University, Kedah, Malaysia

## Abstract

This article aims at providing an insight to the clinical modifications required for the endodontic management of severely dilacerated mandibular third molar. A 35-year-old patient was referred for the root canal treatment of the mandibular left third molar. An intraoral periapical radiograph revealed a severe curvature in both the canals. A wide trapezoidal access was prepared following the use of intermediate-sized files for apical preparation. Owing to increased flexibility, Hero Shaper NITI files were used for the biomechanical preparation and single cone obturation was carried out. Third molars owing to their most posterior location-limited access coupled with a severe curvature pose utmost clinical challenges require meticulous skill, advanced technology, and patience to achieve success.

## 1. Introduction

Endodontic treatments of third molars are considered an ordeal owing to their most posterior location, unpredictable internal anatomy, bizarre occlusal anatomy, and aberrant eruption patterns [1]. Though the extraction of third molars is often the treatment of choice, few clinical situations might demand the retention of these teeth. Third molars might serve as abutments for removable partial denture of fixed prosthesis, where second molars are lost. Moreover, the principle of endodontics is directed at the preservation of each and every functional component of the dental arch [2]. The anatomical variations confronted in third molars range from curved roots, bayonet roots, fused canals, C-shaped canals, etc. The prevalence of curved canals has been found to be relatively higher in mandibular third molars, ranging from 3.3 to 30.92%, compared to maxillary molars that range from 1.33 to 8.46%. Curved root canals pose great difficulty in cleaning, shaping, and obturation of the root canal system with an exponential rise in difficulty with an increase in curvature. Predictability of treatment is ensured from a blend of profound knowledge and meticulous skill of the practitioner. The following article presents a case report of the endodontic treatment of a mandibular third molar with severely curved canals and highlights the various disciplines and modifications employed for its management.

## 2. Case Report

**Figure 1:**
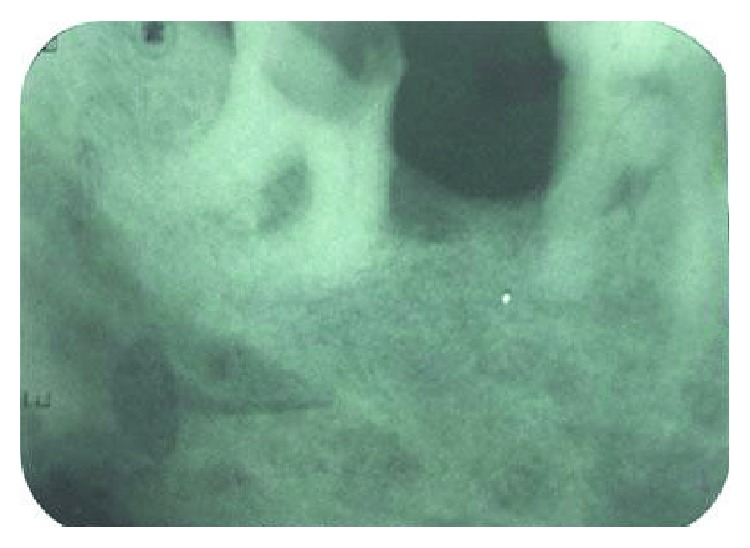
Diagnostic radiograph showing severely curved canals.

A 35-year-old male patient reported to the dental clinic with a history of sharp pain in the left lower back region for the last two days. Clinical examination revealed a deep carious lesion in the left mandibular third molar and a missing left mandibular second molar, extracted two years back. The oral findings were confirmed with an intraoral periapical (IOPA) radiograph depicting a deep carious lesion approaching the pulp in the left mandibular third molar. The IOPA radiograph further revealed curved mesial and distal canals, a sickle-shaped curvature extending from the middle half of the root till apex (Figure 1). Pulp vitality tests (cold and electric pulp test) confirmed the diagnosis of symptomatic irreversible pulpitis. The patient had an intention to restore the missing mandibular second molar; hence, an endodontic treatment was planned for mandibular first and third molar in view of providing a fixed partial denture.

**Figure 2:**
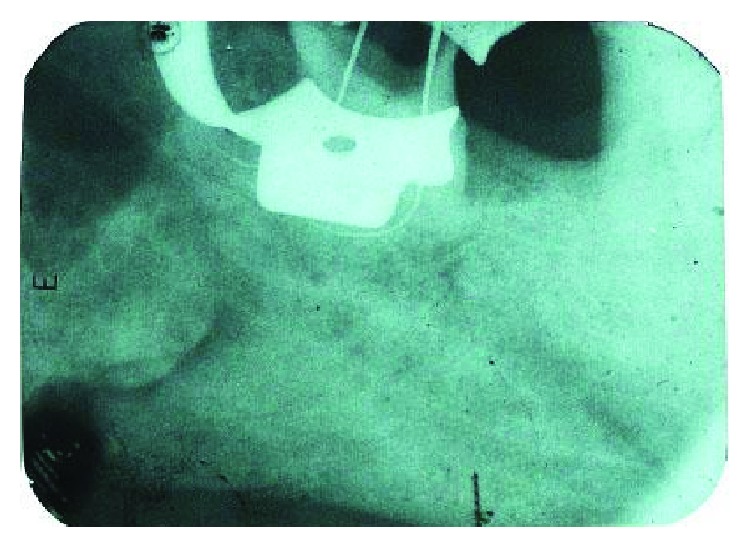
Working length radiograph.

**Figure 3:**
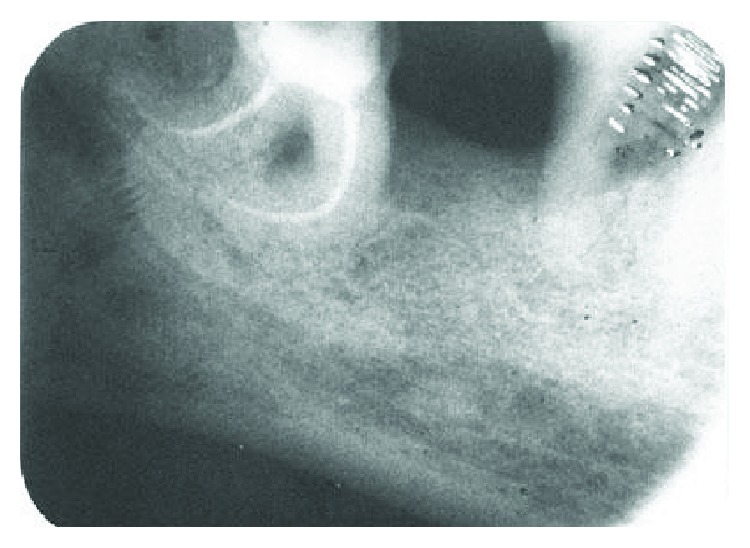
Post obturation radiograph.

**Figure 4:**
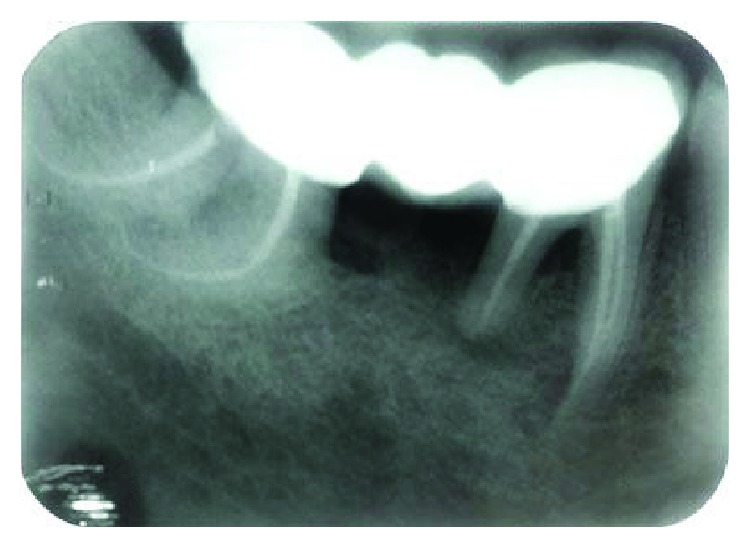
Radiograph showing final restoration.

After adequate local anaesthesia and isolation with a rubber dam, the access cavity was prepared using Endo Access kit (Dentsply) in the mandibular left third molar. After gaining an adequate access, initial scouting of all the root canals was done with K-file no. 10, and the patency of root canals was established. Gates Glidden (GG) drills were placed sequentially in a step-back fashion (i.e., nos. 1, 2, and 3) to allow easy placement of instruments and to gain a straight line access to the apex. The working length was confirmed using an apex locator (Root ZX J. Morita) and SS K-file no. 15 through an IOPA radiograph (Figure 2). Succeeding, path finder files (Dentsply) of intermediate sizes, i.e., no. 13, no. 16, and no. 19, were used in order to closely follow the curvature and maintain the apical spatial orientation. Each filing sequence was accompanied with 17% EDTA (Glyde, Dentsply) followed by copious irrigation with saline and 3.2% NaOCl. The rotary Hero Shaper files were subsequently used in the fashion as instructed by the manufacturer (20-0.6, 20-0.4). Following the biomechanical preparation, the canals were irrigated, flushed with EDTA 17%, and dried prior to obturation. Single cone 4% taper gutta percha cones (Dentsply) were used to obturate all the canals (Figure 3). The post obturation restoration was done with a composite to maintain a good coronal seal (Figure 4). Similarly, endodontic treatment was executed for 36. A three-unit bridge was given to the patient finally.

## 3. Discussion

As a general consensus, endodontic treatment of third molars is avoided and these teeth are doomed for extraction. But for certain clinical situations, the retention of third molars is preferable as where second molars are already missing or need extraction owing to a large carious lesion or third molars are indicated options for transplantations or orthodontic translations. In the aforementioned case, the third molar could serve as an excellent abutment of the fixed prosthesis for restoring the missing second molar. Moreover, the mouth opening and gag reflex of the patient were favourable. Therefore, an endodontic treatment was planned for the concerned third molar.

The posterior-most location of the third molar makes it a clinical dilemma, compromises access of vision and instrumentation, and often presents with bizarre occlusal anatomy and internal patterns. The incidence of curved canals, fused roots, and C-shaped canals is generously reported in the literature. Gulabivala et al. [3] found 10.9% of single-rooted mandibular third molars having C-shaped variants. Hamasha et al. [4] reported the prevalence of dilacerations to be 3.8% and it was the highest in lower third molars, 19.2%. Similarly, the prevalence of curved canals has been found to be relatively higher in mandibular third molars, ranging from 3.3 to 30.92%, compared to maxillary molars that range from 1.33 to 8.46%. A tooth is considered dilacerated when there is a mesial or distal tilt of the root and the angle is equal or exceeds 90 in relation to the tooth or root axis. Another school of thought considers a dilaceration when its apical deviation is equal or exceeds 200 in relation to the normal tooth axis [5].

**Figure 5:**
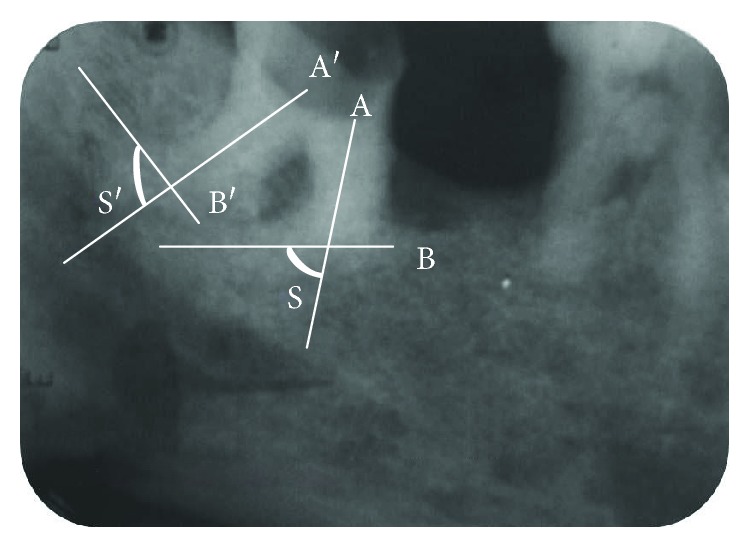
Measurement of Schneider's angle using radiograph. Both *S* and *S*′ are more than 70° (severely curved canals).

Root canal curvatures may be apical, gradual, sickle-shaped, severe-moderate-straight curve, bayonet/S-shaped curve, and dilacerated curve [6]. Curved root canals present as a challenge in cleaning, shaping, and obturation of the root canal system [7]. These curves must always be valued and maintained strictly. The clinical strategy alters with the degree of dilacerations. Various attempts have been made for measuring the extent of curvatures. The most accepted one is given by Schneider. This method involves drawing a line parallel to the long axis of the canal in the coronal third of the root canal and another line drawn from the apical foramina to intersect the first line on a hard copy of the diagnostic radiographic printout. Schneider's angle is formed from the intersection of these lines. Accordingly, the degree of root canal curvature is categorized as straight: 5° or less moderate: 10–20° and severe: 25–70°. Gunday et al. [8] introduced the term “canal access angle” (CAA), another parameter, which provides more information about the coronal geometry of canal curvature. Abiding by Schneider's method, the aforementioned third molar exhibited severe dilacerations (Figure 5) and demanded a cautious preparation at each step. While preparing the curved canals, the following principles were closely followed:

1. To maintain the apical foramen in its original spatial location
2. To gain a straight line access to the site of curvature
3. To respect the anatomical danger zone in curved canals: the inner wall of the middle third and outer aspect of the apical third
4. To use an instrument that closely adapts to the original shape of the canal, respecting its anatomy [9]

The access cavity was prepared using an Endo Access kit (Dentsply). A slight wide tapered access cavity was prepared to allow easy access of instruments. No 10 k file was used as the scouting files as smaller files exhibit sufficient flexibility and control over movement to prepare a glide path. Subsequently, the orifice widening and coronal flaring were achieved using Gates Glidden drill in crown down fashion up to nos. 3, 2, and 1. Guttman [10] suggested preflaring the coronal 1/3 of the canal (at the expense of the tooth structure) to reduce the angle of curvature. Working length was established using file no. 15 and apex locator (Root ZX, Dentsply). This was followed by the usage of intermediate size files, namely, Pathfinder file nos. 13, 17, and 19 (Dentsply). The standardized instruments increase in size by fixed, absolute increments (0.05 mm in diameter, 1.0 mm from the tip end), but increases in size are not constant. For example, there is a 50% increase in size from no. 10 to no. 15 and a 33% increase in size from no. 15 to no. 20, which is tremendous [11]. Such a mismatch of sizes of successive files tends to place excessive force on each file and may lead to the straightening of curvature, “direct perforation,” “ledges” or “false canals,” creation of a “teardrop foramen,” stripping, and blockage of canals. Thus, it becomes imperative to use files of intermediate sizes, precurved files, and anticurvature filing technique.

The final results of the instrumentation of curved root canals may be influenced considerably by the flexibility and diameter of the endodontic instruments. The Hero Shaper nickel-titanium rotary files provide excellent flexibility and centric ability. The canals were prepared using Hero Shaper files up to 20 apical size with a minimal taper of 4%. Copious irrigation was carried out with the repeated use of 3.2% NaOCl and 17% EDTA. NaOCl helps in loosening and flushing out the organic fibrous content, and EDTA manages to dissolve the inorganic matter within the canal [12]. Profuse irrigation was employed to ensure the maintenance of working length and avoid undue debris clogging within the canal. After the final rinse of EDTA and saline, the canals were dried with paper points and obturation was carried out using 4% taper gutta percha cones and a zinc oxide eugenol sealer.

To ensure optimum sealing, the access cavity was sealed with composite restoration. In the subsequent visits, crown preparations were performed and a fixed three-unit prosthesis was delivered to the patient.

## 4. Conclusion

Severely curved canals cannot be an indication for the extraction of a restoratively important third molar. Following the basic principles and taking advantage of new innovations (usage of intermediate precurved sequential filing and flexible rotary systems) in the field of endodontics, even most severely curved canals can be negotiated and treated successfully as in the present case.
